# Exploring the impact of antenatal micronutrients used as a treatment for maternal depression on infant temperament in the first year of life

**DOI:** 10.3389/fnut.2024.1307701

**Published:** 2024-04-22

**Authors:** S. A. Campbell, S. P. Dys, J. M. T. Henderson, H. A. Bradley, J. J. Rucklidge

**Affiliations:** ^1^School of Psychology, Speech and Hearing, University of Canterbury, Christchurch, New Zealand; ^2^Department of Psychology, Simon Fraser University, Burnaby, BC, Canada

**Keywords:** antenatal, prenatal, nutrient, supplement, temperament, infant

## Abstract

Antenatal depression and maternal nutrition can influence infant temperament. Although broad-spectrum-micronutrients (BSM: vitamins and minerals) given above Recommended Dietary Allowances during pregnancy can mitigate symptoms of antenatal depression, their associated effects on infant temperament are unknown. One hundred and fourteen New Zealand mother-infant dyads (45 infants exposed to BSM during pregnancy (range of exposure during pregnancy: 12–182 days) to treat antenatal depressive symptoms (measured by Edinburgh Postnatal Depression Scale) and 69 non-exposed infants) were followed antenatally and for 12 months postpartum to determine the influence of *in utero* BSM exposure on infant temperament. The Infant Behavior Questionnaire–Revised: Very Short-Form assessed temperament at 4 (T1), 6 (T2) and 12 (T3) months postpartum via online questionnaire. Latent growth curve modeling showed BSM exposure, antenatal depression and infant sex did not statistically significantly predict initial levels or longitudinal changes in orienting/regulatory capacity (ORC), positive affectivity/surgency (PAS) or negative affectivity (NEG). Higher gestational age was positively associated with initial PAS, and smaller increases between T1 and T3. Breastfeeding occurrence was positively associated with initial NEG. Although not significant, BSM exposure exerted small, positive effects on initial NEG (*β* = −0.116) and longitudinal changes in ORC (*β* = 0.266) and NEG (*β* = −0.235). While BSM exposure did not significantly predict infant temperament, it may mitigate risks associated with antenatal depression. BSM-exposed infants displayed temperamental characteristics on par with typical pregnancies, supporting the safety of BSM treatment for antenatal depression.

## 1 Introduction

The antenatal environment substantially impacts fetal development, with research showing maternal behavior and emotional states during pregnancy influences fetal programming (1, 2). As a consequence, maternal psychiatric status has become a central component of antenatal care, particularly with respect to understanding its effect on long term social, emotional and behavioral infant development (3). Initially proposed to explain the relation between maternal antenatal health and the emergence of later diseases in offspring (4), the fetal programming hypothesis has been applied to behavioral and psychological development of infants, notably infant temperament (5).

Over the past three decades, several definitions and approaches on the development of temperament have been proposed (6). Despite their differences, researchers have agreed that temperament: (1) is not a trait, rather a collection of traits, (2) can be thought of as behavioral tendencies rather than specific behaviors, (3) is biologically based, (4) refers to individual differences, and (5) can be shaped through experience (7, 8). One widely accepted definition describes temperament as “constitutional differences in reactivity and regulation influenced over time by heredity, maturation, and experience” (9). This emphasizes the combination between biology and environment: an individual’s temperament is genetically influenced and thus relatively stable; however, it is still shaped by the environment individuals develop in and interact with over time. Infant temperament has been positively associated with later social competence (10), identified as a risk factor in the development of future psychopathology including ADHD (11) and externalizing/internalizing behavioral problems (e.g., anxiety, depression) (12), and is often considered the building blocks of adult personality (13). Given this understanding of how temperament originates and its impact on long term development, it is not surprising the connection among temperament, fetal programming, and antenatal depression is being increasingly explored.

Thomas and Chess (14) initially proposed nine dimensions to measure and explain traits of infant temperament. Over time, these dimensions have been altered through factor analysis and investigators have determined that temperament could be broadly measured over three dimensions: (1) negative affectivity (NEG), (2) positive affectivity/surgency (PAS) and; (3) orienting/regulatory capacity or effortful control (ORC) (15). NEG includes displays of typically negative behaviors, e.g., sadness, fear, distress to limitations; PAS contains typically positive behaviors, e.g., approach, smiling and laughter; and ORC includes regulatory functioning, e.g., orienting, soot ability, cuddliness (15, 16).

Detangling the effects of antenatal depression from the effects of postpartum depression is complex, as many studies fail to separate perinatal depression into two distinct periods, antenatal or postnatal. The existing limited evidence suggests that antenatal depression is associated with, and may even predict, aspects of infant temperament, most notably negative affectivity, which is of particular importance given the dyadic nature of the mother-infant relationship and the impact affect has in transactional processes within the wider family system (17). Antenatal depression has been associated with increased risk of infant irritability and fussiness (17), as a predictor of emotional reactivity (18), and negative affectivity characterized by a lack of smiling, difficulty soothing, and increased sadness (19–21). Infant negative affectivity has been implicated as a risk factor for future psychopathology (21, 22).

Five systematic reviews have examined the association between antenatal maternal mental disorders and infant temperament (5, 19, 23–25). These reviews provide conflicting results, with four suggesting antenatal depression was associated with difficult or more negative temperament (19, 23–25), and the other concluding the evidence was equivocal (5).

Rouse and Goodman (17) identified that the timing of exposure to antenatal depression is an important variable influencing infant temperament, suggesting a window of vulnerability in mid pregnancy, while two other studies have found the impact of antenatal depression on infant negative affectivity was moderated by genetic factors (26, 27) indicating an interaction among maternal psychiatric status, genetics, and infant temperament.

Given the negative effects of maternal depression on infant temperament such as increased displays of negative affectivity (21) and emotional reactivity (28), it is expected that treating depression during pregnancy may mitigate these negative effects on the infant. Current treatment recommendations include psychological treatments for mild to moderate antenatal depression, such as cognitive behavioral therapy (CBT) and interpersonal psychotherapy (IPT) (29), with some evidence for a small but positive effect on offspring outcomes, although findings on these benefits are inconsistent (30, 31). However, women often do not access these treatments due to issues with time, cost, stigma, and childcare issues. As far as we are aware, there are no studies that have explored the effect of psychological treatments for antenatal depression specifically on infant temperament.

For more severe depression antidepressant medication (AD), such as selective serotonin reuptake inhibitors (SSRI) or selective norepinephrine reuptake inhibitors (SNRI) are recommended (29, 32). The effects of AD use in pregnancy on anthropometric outcomes have been explored, with some observational studies suggesting an increased risk of preterm birth (33), and reduced birth weight (34). While negative effects may be transient, and with preliminary findings suggesting ADs given antenatally do not appear to exert significant effects on temperament (35), the scarcity of research and no RCTs exploring the effect of medication use in the pregnant population, makes the safety of ADs in the long-term difficult to determine. Indeed, there is some hesitancy with continued use of ADs within the pregnant population (36) and psychiatric medication use can reduce by 80 percent during pregnancy (37), highlighting the importance of a careful risk–benefit analysis as well as the need for more research on alternative treatment options in pregnancy, and their subsequent effects on infant outcomes.

Growing attention is being given to the intrauterine nutritional environment, particularly improving maternal nutrient status during pregnancy (38) as the body’s nutritional requirements increase to support the metabolic and hormonal changes of the mother and growth and development of the fetus. As a result of the increased nutritional demand, it is likely that many pregnant people are vulnerable to inadequate nutrient intake (39), thus supplementation with vitamins and minerals have become commonplace in obstetric care (40).

The effects of poor nutrition during pregnancy has been extensively explored, particularly given the outcome of The Dutch Famine Birth Cohort Study, where *in-utero* undernutrition was predictive of future psychopathology (41). Since then, numerous studies have documented the effects of dietary intake on infant outcomes (42–46), although only three in the past decade focused on infant temperament (47–49), with higher adherence to healthier diets being associated with higher scores on temperament dimensions of positive affectivity and orienting/regulatory capacity.

A newer line of research is investigating the effects of supplementation with vitamins and minerals (broad spectrum micronutrients or BSM) on antenatal depression, based on extensive studies showing that BSM can positively impact on symptoms of depression in non-pregnant populations (50). Although several of the interventions were conducted within physically and psychologically well populations, participants who experienced psychological distress or severe physical illness tended to improve more with nutritional supplementation compared to participants who were well (50), thus providing support for BSM as a treatment option, which could extend into pregnant populations.

As far as we are aware, there is no literature on the relation between antenatal nutrient supplementation with BSM and infant temperament; however, there is significant evidence for the benefits of nutrient supplementation in pregnancy for overall infant development (51–53). The effects of antenatal supplementation with single nutrients such as folic acid, iron and iodine on infant outcomes although mixed, report improvements in birth outcomes (53), cognitive and motor performance in the first year (54, 55) and reduced behavioral problems later in life (56, 57). Despite these improvements, there are some reports of detrimental effects to infant outcomes related to excessive supplementation with one nutrient given over the recommended dietary allowance (58–60). Further, where no associations have been found, there are also no adverse effects reported for infant outcomes (61) suggesting that with careful monitoring of dosage, the potential benefits to infant development could outweigh the potential risks.

Supplementation with multiple micronutrients, although limited, has been found to be superior to single nutrient and iron+folic acid/iodine+folic acid supplements for improving birth outcomes (62), cognitive and motor development at 7 months (63) and increased scores of communication, motor and social skills at 36 months old (64). Multiple nutrients are known to work in combination with each other to exert their effect rather than in isolation, providing a potential explanation for this observed superiority over single-nutrient supplementation (65).

Despite the reported association between antenatal depression and infant temperament (17, 19–25), the specific mechanisms of the association remain inconclusive. Negative affectivity and poor regulatory capacity have been strongly associated with maternal antenatal mood state (26, 66, 67). Thus, targeting antenatal depression may improve maternal mental health thereby resulting in a chain of biological and environmental changes which could positively impact infant temperament and developmental outcomes.

Healthier dietary patterns in pregnancy have been associated with improvements in infant affectivity and regulatory capacity, characteristic of an “easier” infant temperament and although not directly comparable to diet studies, improving maternal nutritional status via supplementation may have similar effects on infant temperament. Several nutrients contained within the BSM formula used in the current study are known co-factors required for the synthesis of serotonin, a neurotransmitter linked to emotion regulation (68). It is possible that increasing maternal concentrations of vital nutrient co-factors in pregnancy may influence both maternal and fetal serotonin production (69), impacting emotion regulation a key component of temperament.

The current study aimed to identify whether BSM supplementation given above the Recommended Dietary Allowance but typically below the Tolerable Upper Level (the highest level of daily nutrient intake that is likely to pose no risk of adverse health effects to almost all individuals in the general population) in a sample of pregnant women with antenatal depression was associated with any adverse risk to infant temperament (such as high negative affectivity or low regulatory capacity, characteristics of a more difficult temperament) (48), or differences in initial levels or developmental changes in infant temperament dimensions (NEG, PAS and ORC) across the first year of life.

Given the existing literature finding a positive association between healthier maternal nutrition and infant temperament, and the evidence for BSM as a treatment for improving psychiatric symptoms, we hypothesized BSM exposure would pose no adverse risk to infant temperament, predict higher initial levels of positive temperament behaviors (ORC and PAS) and be associated with developmental changes on par or better than non-exposed infants on measures of temperament across the three time points; specifically with lower scores on NEG, and higher scores of PAS and ORC on the IBQ-R:VSF.

## 2 Methods

**Figure 1:**
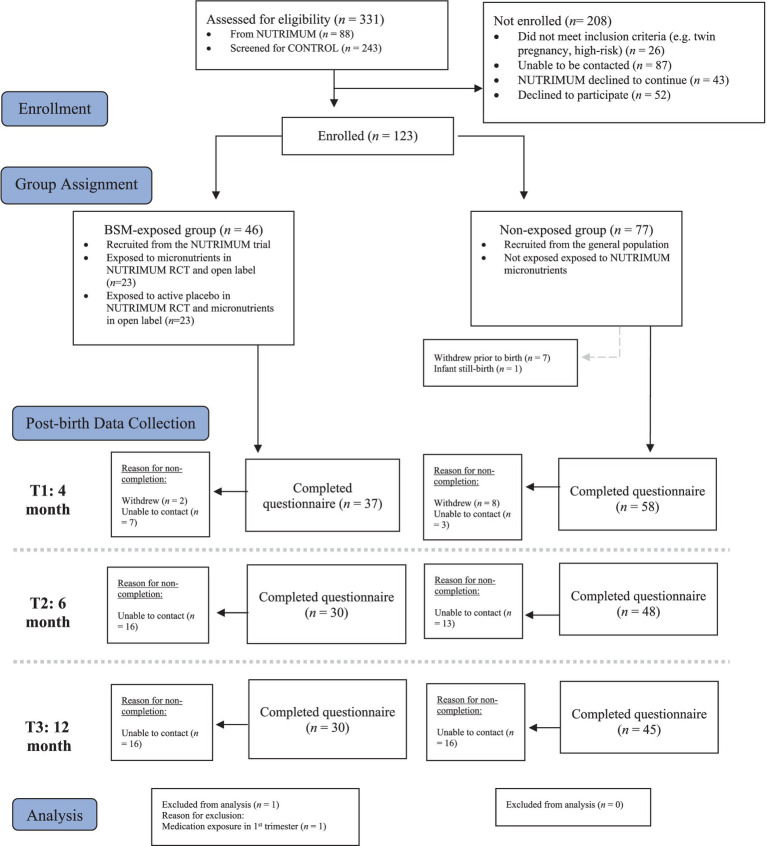
Group sample sizes during longitudinal data collection, reasons for non-completion of questionnaires and exclusions from data analysis.

In this longitudinal follow up study, a sample of 123 infants were followed for 12 months in Aotearoa, New Zealand. A final sample of 114 mother-infant dyads were included in data analysis. Further information on detailed grouping and flow of participants during data collection, reasons for non-completion and exclusions from data analysis are shown in Figure 1.

A portion of the current sample included 46 infants whose mothers had previously participated in a randomized, placebo-controlled trial (RCT) conducted between 2017 and 2021 (NUTRIMUM Trial: (70, 71)). For the RCT, participants between 12 and 24 weeks’ gestation identified with depressive symptomology during pregnancy (Edinburgh Postnatal Depression Scale: EPDS ≥13), and not taking any psychiatric medication, were randomized to receive daily BSM or a placebo for 12 weeks during their pregnancy The BSM formula used in the NUTRIMUM study, Daily Essential Nutrients (DEN), contains 36 essential vitamins, minerals, amino acids and antioxidants, and this combination of nutrients has been explored as a treatment for other psychiatric illnesses in non-pregnant populations (65). For the full list of ingredients contained within DEN, see Table 1. The RCT phase was followed by an open-label phase of BSM until the birth of the infant, providing an opportunity for naturalistic observation of infant temperament in a micronutrient exposed group of infants.

**Table 1:** Ingredients of micronutrient (BSM) intervention from the NUTRIMUM trial.

Daily Essential Nutrients Supplement Facts	
Total dose (4 capsules, 3 times daily)	
Vitamin A (as retinyl palmitate)	5,760 IU
Vitamin C (as ascorbic acid)	600 mg
Vitamin D (as cholecalciferol)	3,000 IU
Vitamin E (as d-alpha tocopheryl succinate)	360 IU
Vitamin K (75% as phylloquinone; 25% as menaquinone-7)	120 mcg
Thiamin (as thiamin mononitrate)	60 mg
Riboflavin	18 mg
Niacin (as niacinamide)	90 mg
Vitamin B6 (as pyridoxine hydrochloride)	69.9 mg
Folate (as L-methylfolate calcium)	801 mcg
Vitamin B12 (as methylcobalamin)	900 mcg
Biotin	1,080 mcg
Pantothenic acid (as d-calcium pantothenate)	30 mg
Calcium (as chelate)	1,320 mg
Iron (as chelate)	13.8 mg
Phosphorus (as chelate)	840 mg
Iodine (as chelate)	204 mcg
Magnesium (as chelate)	600 mg
Zinc (as chelate)	48 mg
Selenium (as chelate)	204 mcg
Copper (as chelate)	7.2 mg
Manganese (as chelate)	9.6 mg
Chromium (as chelate)	624 mcg
Molybdenum (as chelate)	144 mcg
Potassium (as chelate)	240 mg

Proprietary blend: Choline bitartrate, Alpha-lipoic acid, Mineral wax, Inositol, Acetyl-L-carnitine, Grape seed extract, Ginkgo biloba leaf extract, L-methionine, N-acetyl-L-cysteine, Boron (as chelate), Vanadium (as chelate), Lithium orotate (as chelate), Nickel (as chelate). Other ingredients: Vegetarian capsule (hypromellose), Microcrystalline cellulose, Magnesium stearate, Silicon dioxide, Titanium dioxide.

Infants born to participants enrolled in the NUTRIMUM trial (BSM-exposed group) were either exposed to micronutrients during *both* the RCT phase and the open label phase or exposed to the active placebo during the RCT phase and only exposed to micronutrients in the open label phase. Additionally, given that mothers could be between 12 to 24 weeks gestation when they started the RCT, this resulted in varying days of possible exposure to the micronutrients *in-utero*, from zero days up to 196 days.

For example, if a participant entered the study at 19 weeks’ gestation and was randomized to the active placebo group during RCT and gave birth at 40 weeks’ gestation, they would enter the open label phase at 31 weeks’ gestation and micronutrient exposure would be 70 days. However, if a participant entered the study at 12 weeks’ gestation and was randomized to the micronutrient group during RCT, and gave birth at 36 weeks’ gestation, they would enter the open label phase at 24 weeks’ gestation and micronutrient exposure would be 168 days.

The NUTRIMUM trial was prospectively registered: Australian and New Zealand Clinical Trials Registry; ACTRN12617000354381, and the overall study received ethical approval from relevant university and national ethical review boards.

The remainder of the sample (*n* = 77) was recruited from the general population and included infants born to mothers not receiving the NUTRIMUM trial supplement although could be experiencing mood symptoms (measured on a continuum) or being treated for antenatal mood symptoms with antidepressants (SSRI: *n* = 21; SNRI: *n* = 3). Current nutrient supplementation status was collected at study entry: 71.8% of the sample not enrolled in the NUTRIMUM trial (non-exposed group) reported taking a daily nutrient supplement (e.g., folic acid, iodine, B vitamins, pregnancy multivitamin), below the Recommended Dietary Allowance (RDA), and significantly lower doses than those provided to the MN group as part of the NUTRIMUM trial.

Inclusion criteria for all participants: (1) pregnant and between 12–34 weeks’ gestation, (2) aged ≥16 years, and (3) a low-risk singleton pregnancy. Exclusion criteria for all participants included: (1) women with pregnancy complications or high-risk pregnancy (e.g., placenta previa, preeclampsia), (2) known fetal abnormalities, (3) serious current or historical medical condition (e.g., hypertension, kidney disease), (4) known metabolic conditions (e.g., Wilson’s disease, hemochromatosis), and (5) untreated or unstable thyroid disease and known neurological disorders (e.g., epilepsy, multiple sclerosis, narcolepsy). Initial recruitment was confined to participants residing in Canterbury, New Zealand; however, due to the COVID-19 lockdown in March 2020, the study was adapted to work remotely, and enrolments were opened to anyone residing anywhere in New Zealand who met initial inclusion criteria (*n* = 7).

## 3 Procedure

Eligible participants were invited to attend an initial appointment either at the study site or via telephone/video call, where the study was explained and written informed consent was obtained. Individuals who screened but not eligible were informed via email and provided links to support services and encouraged to contact their GP or lead maternity carer for any additional psychological support.

Enrolled participants were monitored throughout pregnancy via online questionnaires every 4 weeks using Qualtrics Survey software. The BSM exposed group were monitored at a higher frequency (every 2 weeks) via online questionnaires and met with a clinician either in-person or via telephone/video call every 4 weeks as part of their enrolment in the NUTRIMUM Trial to monitor mood and any potential side effects of the RCT intervention. After birth, all participants completed questionnaires at 4 and 6 months postpartum, either completed at the study site or online via email link for those who did not live locally. Participants who traveled to the study site received a NZ$10 petrol voucher for each visit to cover travels costs. At 12 months postpartum, participants were sent an email link to an online questionnaire and upon completion, received a $20 petrol voucher via mail to thank them for their time.

## 4 Measures

### 4.1 Primary measure

Maternal perceptions of infant temperament was assessed using the Infant Behavior Questionnaire–Revised: Very Short Form (IBQ-R:VSF) (15), which is a 37-item self-report questionnaire based on the Infant Behavior Questionnaire–Revised (IBQ-R) (16). It contains three subscales: *PAS* (e.g., How often during the last week did the baby smile or laugh when given a toy?), *NEG* (e.g., When you were busy with another activity, and your baby was not able to get your attention, how often did s/he cry?) and *ORC* (e.g., When showing the baby something to look at, how often did s/he soothe immediately?).

The Infant Behavior Questionnaires (IBQ) (72) are the most widely used measure of infant temperament (15) and the revised very short version (IBQ-R:VSF) was most appropriate given it was originally developed for use in longitudinal studies and is suitable for repeated measures and time-sensitive administration (15).

Mothers completed the IBQ-R:VSF at 4-, 6- and 12-months where they were asked the frequency of specific behaviors over a seven-day period. Each question was answered on an eight-point scale with responses ranging from (1) never to (7) always. In the event certain behaviors did not arise within the past week, a “does not apply” option was available. Responses for each of the three subscales were averaged, and interpreted on a continuum, with higher scores indicating greater display of that temperament dimension. The internal consistency of the IBQ-R:VSF scales is between 0.70 to 0.92 (15).

### 4.2 Additional measures

Information about maternal mental health and nutritional status was collected via online questionnaires at study entry, throughout pregnancy and post-birth. Information about infant anthropometric characteristics (e.g., gestational age, weight) was obtained through hospital records. Full details and references on measures used are explained elsewhere (70).

The Edinburgh Postnatal Depression Scale (EPDS) has shown strong validity for use in measuring depressive symptoms during pregnancy and the postpartum period (73, 74) and has a Cronbach’s alpha of 0.83, indicating good internal consistency (75). A cutoff of 13 was used to identify the presence of moderate depressive symptoms (75). An average antenatal depression score was calculated for each participant based on monthly EPDS scores collected during pregnancy.

A variable was created to determine the occurrence of breastfeeding postnatally. Participants were grouped (lowest to highest level of occurrence) based on whether they had never breastfed, breastfed on and off (e.g., used a combination of breast and formula feeding) or exclusively breastfed. Breastfeeding occurrence has previously been associated with temperament.

### 4.3 Statistical analysis

Latent growth curve modeling using MPlus 8 was used to determine changes in temperamental outcomes across time. To start, we screened for univariate outliers, with criteria set to absolute values of skew <2 and kurtosis <7 (76). We examined whether micronutrient exposure was related to children’s temperamental development using latent growth curve (LGC) modeling (77) in two main stages. In the first stage, we identified normative patterns of development for each temperament outcome (i.e., NEG, ORC and PAS) from T1 to T3 (T1: 4 month; T2: 6 month; T3: 12 month) by modeling two latent factors representing the initial status (i.e., intercept) and longitudinal change (i.e., slope). We identified the best fitting unconditional models by comparing three nested models using the χ^2^ difference test. The models we compared were: (a) stability only model, wherein we only estimated an intercept factor, (b) linear change model, wherein we added a slope factor and fix loadings to 0, 1, and 4 (to account for the unequal spacing between timepoints—i.e., 4, 6, and 12 months), and (c) a nonlinear change model, wherein we freely estimated the T2 factor loading. In the second step, we ran a conditional growth curve model, which included our independent variables: micronutrient exposure as our focal predictor, as well as gestational age at birth, infant sex (0 = female; 1 = male), mean antenatal depression and breastfeeding occurrence as our control variables.

Model fit was evaluated using standard indices (78, 79). We considered the following criteria as reflective of acceptable fit: a non-significant chi-square test, a comparative-fit-index (CFI) and Tucker-Lewis-Index (TLI) > 0.90, root-mean-square-error-of-approximation (RMSEA) < 0.08 with 90% confidence intervals (CI), standardized root mean square residual (SRMR) < 0.08. Analyses were run using Mplus 8 (80) using maximum likelihood estimation of the parameters (ML). We handled missing data using Full Information Maximum Likelihood (FIML) estimation because it is preferable to traditional approaches (e.g., listwise deletion, mean substitution) which have been shown to reduce power, underestimate variability, undermine the validity of sample characteristics, or a combination thereof (81, 82). However, listwise deletion was used to handle participants who provided no data at any of the timepoints of interest. We ran Little’s MCAR test to evaluate whether our data were missing completely at random (MCAR), which would suggest that our missing data could be estimated reasonably using observed data.

## 5 Results

### 5.1 Sample characteristics

Mean maternal age was 31.4 years, and 77.2% European. Mean length of exposure to BSM was 104 days (*SD* = 44.17; range = 12–182 days). Mean infant gestational age was 39.4 weeks (*SD* = 1.5). Further sample information can be found in Table 2.

**Table 2:** Maternal demographic characteristics at study entry*.

	Full sample (*N* = 114)		BSM-exposed (*n* = 45)		Non-exposed (*n* = 69)		Group effect	
	*n* / *M*	% / *SD*	*n* / *M*	% / *SD*	*n* / *M*	% / *SD*	*χ*^2^/*η*_p_^2^	*p*
Maternal ethnicity							9.78	0.082
NZ Māori	7	6.1	2	4.4	5	7.2		
Pacific Island (Tongan, Samoan, Fijian, Niuean)	3	2.6	2	4.4	1	1.4		
Asian	6	5.3	5	11.1	1	1.4		
MELAA	7	6.1	3	6.7	4	5.8		
European (New Zealand, British, Australia, Italian)	88	77.2	30	66.7	58	84.1		
Household income							2.14	0.344
Low ($0 - $39,999)	19	16.7	10	22.2	9	13		
Middle ($40,000 - $79,999)	39	34.2	16	35.5	23	33.3		
High ($80,000+)	56	49.1	19	42.2	37	53.6		
Maternal background characteristics								
Young mother (< 21 years)	3	2.6	1	2.2	2	2.9	0.049	0.825
Single parent family	8	7.0	4	8.9	4	5.8	0.399	0.528
Ethnic minority	21	18.4	11	24.4	10	14.5	1.79	0.180
Low educational qualification	16	14.0	8	17.8	8	11.6	0.863	0.353
Low SES (NZSEI-13)	9	7.9	4	8.9	5	7.2	0.101	0.751
Total social risk score (M)	0.5	0.7	0.6	0.7	0.4	0.7	0.020	0.137
Maternal clinical characteristics								
Age (M)	31.4	4.6	32.1	4.7	30.9	4.6	0.016	0.177
SES status (NZSEI-13) (M)	56.9	16.8	54.8	17.6	58.3	16.3	0.010	0.290
Pregnancy Alcohol use	21	18.4	8	17.8	13	18.8	0.021	0.886
Current Smoker	3	2.6	0	0	3	4.3	2.01	0.156
Pregnancy Drug use	5	4.4	2	4	3	4.3	0.001	0.980
Maternal antenatal wellbeing (at study entry)								
EPDS score	11.9	6.4	16.5	2.71	8.9	6.35	0.337	**<0.001**
GAD-7	7.1	5.0	8.8	4.2	6.1	5.15	0.072	0.004
PSS ^a^	17.6	7.2	21.6	4.8	15.1	7.4	0.193	**<0.001**
DASS-21								
Depression	8.9	7.6	12.4	7.2	6.6	7.0	0.142	**<0.001**
Anxiety	6.5	5.8	6.9	5.9	6.2	5.8	0.004	0.530
Stress	14.2	8.0	17.1	6.1	12.3	8.5	0.087	**0.001**
Nutrition score (DST) (M)^b^	66.5	9.4	66.2	7.5	66.8	10.5	0.001	0.733
Not at risk (n, %)^b^	12	10.5	2	4.55	10	14.3		
Possible risk (n, %)^b^	77	67.5	34	75.5	43	62.3		
At risk (n, %)^b^	23	20.2	9	20.45	14	20.0		
Mean EPDS score through pregnancy	8.6	4.2	9.5	2.5	8.0	4.9	0.032	0.058
Infant clinical characteristic (at birth)								
Female sex	54	47.4	18	40	36	52.17	1.62	0.203
Gestational age (M)	39.4	1.5	39.6	1.3	39.3	1.7	0.010	0.298

MN, exposed to BSM in-utero; CONTROL, not exposed to BSM in-utero; SES, socioeconomic status; NZSEI-13, New Zealand socioeconomic status index 2013; EPDS, Edinburgh Postnatal Depression Scale (≥13 is presence of moderate depressive symptoms; GAD-7, Generalized Anxiety Scale; PSS, Perceived Stress Scale; DASS, Depression, Anxiety, Stress Scale). *This table indicates demographics for mother-infant dyads that were still currently active at the time of infant birth, which is considered the beginning of the postpartum period; a, missing data; MN (*n* = 43); b, missing data; Control (*n* = 67). The Generalized Anxiety Scale (GAD-7) is a self-report measure used to identify generalized anxiety symptoms over the past 2 weeks; scores range from 0 to 21, with higher scores identifying more severe symptoms. The Perceived Stress Scale (PSS) is a self-report measure used to identify an individual’s perceived level of stress over the past 4 weeks. Scores range from 0 to 40 (with higher scores indicating higher stress). The Depression, Anxiety, Stress scale (DASS-21) is a 21-item self-report measure with three subscales, each measuring the severity of depression, anxiety and stress ranging from normal to extremely severe. Each scale has a maximum score of 21, with elevated scores indicating more severe symptoms on that domain. The Dietary Screening Tool (DST) was adapted for a New Zealand population and used to collect information on nutritional status and dietary intake at study entry. It has a total score of 100, with higher scores indicating a healthier dietary pattern and scores below 60 indicating at nutritional risk.

### 5.2 Orienting/regulatory capacity

For orienting/regulatory capacity, the best fitting unconditional model was the linear change model, which fit the data well χ^2^ (1) = 1.175, *p* = 0.278, *CFI* = 0.997, *RMSEA* = 0.041 (90% [CI = 0.000, 0.269]), *SRMR* = 0.019. The variance (*s*^2^ *= 0*.289, *p = 0*.000) of the intercept was significant, suggesting that participants started with different initial levels of orienting/regulatory capacity at T1. The mean of the slope revealed a decrease on orienting/regulatory capacity from T1 to T3 (*M* = −0.069, *p =* 0.001), while the variance on the slope was not significant (*s*^2^ *= 0*.023, *p = 0*.081) indicating although there was an overall group decrease in orienting/regulatory capacity over time, there were not significant interindividual differences in how participants’ scores varied across time.

Next, we tested a conditional model assessing whether micronutrient exposure predicted the intercept or the slope of orienting/regulatory capacity (results displayed in Table 3). The conditional model fit the data well χ^2^(6) = 4.519, *p* = 0.606, *CFI* = 1.000, *RMSEA* = 0.000 (90% CI [0.000, 0.109]), SRMR = 0.060). BSM exposure did not predict initial levels of orienting/regulatory capacity (*b =* −0.001, β = −0.057, *p = 0*.626). Infant sex (*b = 0*.227, β = 0.179, *p = 0*.065), gestational age at birth (*b =* −0.025, β = −0.058, *p = 0*.611), breastfeeding occurrence (*b = 0*.142, β = 0.143, *p = 0*.197) and mean antenatal depression (*b =* −0.018, β = −0.115, *p = 0*.216) also did not predict the intercept. Similarly, BSM exposure did not significantly predict longitudinal changes in orienting/regulatory capacity, though the effect was small and in the expected direction (*b = 0*.001, β = 0.266, *p = 0*.060). Infant sex (*b =* −0.064, β = −0.227, *p = 0*.085), gestational age at birth (*b =* −0.016, β = −0.169, *p = 0*.158), breastfeeding occurrence (*b =* −0.036, β = −0.163, *p = 0*.231), and mean antenatal depression (*b =* −0.003, β = −0.097, *p = 0*. 433) also did not predict the slope of orienting/regulatory capacity. The model accounted for a moderate part of the variance of the intercept (*R*^2^ = 0.063) and the slope (*R*^2^ = 0.164) of orienting/regulatory capacity behavior.

**Table 3:** Conditional latent growth curve model—orienting/regulatory capacity.

	Intercept			Slope		
	*b*	β	*p*	*b*	β	*p*
BSM Exposure	−0.001	0.057	0.626	0.001	0.266	0.060
Gestational age	−0.025	−0.058	0.611	−0.016	−0.169	0.158
Infant sex	0.227	0.179	0.065	−0.064	−0.227	0.085
Mean antenatal depression	−0.018	−0.115	0.216	−0.003	−0.097	0.433
Breastfeeding occurrence	0.142	0.143	0.197	−0.036	−0.163	0.231

### 5.3 Positive affectivity/surgency

For positive affectivity/surgency, the best fitting unconditional model was the nonlinear change model, which fit the data well, χ^2^(1) = 0.216, *p* = 0.642, *CFI* = 1.000, *RMSEA* = 0.000 (90% CI [0.000, 0.203]), *SRMR* = 0.045. In this model, the variance (*s*^2^ *= 0*.787, *p = 0*.000) of the intercept was significant, suggesting that participants started with different initial levels of positive affectivity/surgency at T1. The mean and variance of the slope were significant, with the slope revealing an increase in positive affectivity/surgency from T1 to T3 (*M* = 0.283, *p = 0*.000) and the variance (*s*^2^ *= 0*.036, *p = 0*.000) indicating significant interindividual differences in how participants’ scores varied on positive affectivity/surgency across time.

We then tested a conditional model with the same predictor and control variables as above, to see whether they were predictive of the intercept or slope (results displayed in Table 4). The conditional model fit the data well χ^2^(6) = 7.436, *p* = 0.282, *CFI* = 0.978, *RMSEA* = 0.048 (90% CI [0.000, 0.144]), *SRMR* = 0.045). Gestational age at birth (*b* = 0.102, β *= 0*.171, *p = 0*.048) significantly predicted intercept, indicating higher gestational age was associated with a higher initial level of positive affectivity/surgency behavior. BSM exposure (*b =* −0.001, β = −0.091, *p* = 0.367) did not predict initial levels of positive affectivity/surgency. Infant sex (*b =* 0.207, β = 0.116, *p* = 0.259), breastfeeding occurrence (*b =* 0.078, β = 0.056, *p* = 0.566), and mean antenatal depression (*b =* −0.002, β = −0.010, *p* = 0.925) also did not predict the intercept. Similarly, BSM exposure did not predict longitudinal changes in positive affectivity/surgency *(b = 0*.000, β = 0.008, *p* = 0.943). Infant sex *(b =* −0.038, β = −0.095, *p* = 0.366), breastfeeding occurrence (*b =* −0.013, β = −0.042, *p = 0*.717), and mean antenatal depression (*b = 0*.002, β *= 0*.035, *p = 0*.735) also did not predict the slope of positive affectivity/surgency. Gestational age at birth (*b =* −0.041, β *=* −0.311, *p = 0*.000) significantly predicted the slope positively, indicating that infants with a higher gestational age at birth showed lower intraindividual increases in positive affectivity/surgency over time. The model accounted for a moderate part of the variance of the intercept (*R*^2^ = 0.055) and the slope (*R*^2^ = 0.121) of positive affectivity/surgency behavior.

**Table 4:** Conditional latent growth curve model—positive affectivity/surgency.

Positive affectivity/surgency	Intercept			Slope		
	*b*	β	*p*	*b*	β	*p*
BSM Exposure	−0.001	−0.091	0.367	0.000	0.008	0.943
Gestational age	**0.102**	**0.171**	**0.048**	**−0.041**	**−0.311**	**0.000**
Infant sex	0.207	0.116	0.259	−0.038	−0.095	0.366
Mean antenatal depression	−0.002	−0.010	0.925	0.002	0.035	0.735
Breastfeeding occurrence	0.078	0.056	0.566	−0.013	−0.042	0.717

For bolded parameters, *p* < 0.05.

### 5.4 Negative affectivity

Finally, we tested the unconditional models for negative affectivity. The linear change model fit the data well, χ^2^(1) = 0.028, *p* = 0.868, *CFI* = 1.000, *RMSEA* = 0.000 (90% CI [0.000, 0.138]), *SRMR* = 0.004. In this model, the variance (*s*^2^  *= 0*.511, *p = 0*.000) of the intercept was significant, suggesting that participants started with different initial levels of negative affectivity at T1. The mean of the slope revealed an increase on average negative affectivity from T1 to T3 (M = 0.123, *p* = 0.000); however, the variance on the slope was not significant indicating there were not significant differences between participants in the overall increase (*s*^2^ *= 0*.015, *p = 0*.608).

In the conditional model for negative affectivity, we included the same control variables as in previous models to determine if they predicted the slope or intercept of negative affectivity (results displayed in Table 5). The conditional model fit the data well χ^2^(6) = 2.347, *p* = 0.885), *CFI* = 1.000, *RMSEA* = 0.000 (90% CI [0.000, 0.061], *SRMR* = 0.022 (results displayed in Table 3). BSM exposure did not predict initial levels of children’s negative affectivity (*b =* −0.001, β *=* −0.116, *p = 0.*368). Infant sex (*b = 0*.007, β *= 0*.005, *p = 0*.971), gestational age at birth (*b =* −0.013, β *=* −0.026, *p = 0*.807), and mean antenatal depression (*b = 0*.028, β *= 0*.163, *p = 0*.197), also did not predict the intercept. Breastfeeding occurrence (*b = 0*.248, β *= 0*.220, *p = 0*.045) significantly predicted the intercept, indicating higher occurrences of breastfeeding were associated with higher initial levels of negative affectivity. BSM exposure (*b = 0*.000, β *=* −0.235, *p = 0*.490) did not significantly predict longitudinal changes in negative affectivity. Infant sex (*b = 0*.050, β *= 0*.250, *p = 0*.597), gestational age at birth (*b = 0*.008, β *= 0*.118, *p = 0*.653), breastfeeding occurrence (*b = 0*.039, β *= 0*.245, *p = 0*.553), and mean antenatal depression (*b = 0*.006, β *= 0*.243, *p = 0*.508), also did not predict the slope of negative affectivity. The model accounted for small part of the variance of the intercept (*R*^2^ = 0.096) and the slope (*R*^2^ = 0.222) of negative affectivity.

**Table 5:** Conditional latent growth curve model—negative affectivity.

	Intercept			Slope		
	b	β	*p*	b	β	*p*
BSM Exposure	−0.001	−0.116	0.368	0.000	−0.235	0.490
Gestational age	−0.013	−0.026	0.807	0.008	0.118	0.653
Infant sex	0.007	0.005	0.971	0.050	0.250	0.597
Mean antenatal depression	0.028	0.163	0.197	0.006	0.243	0.508
Breastfeeding occurrence	**0.248**	**0.220**	**0.045**	0.039	0.245	0.553

For bolded parameters, *p* < 0.05.

## 6 Discussion

Antenatal depression is a significant public health issue, and the limited treatment options available have significant limitations with respect to infant outcomes. Untreated, antenatal depression is associated with a more difficult temperament in the infants which is a risk factor for future psychopathology. For these reasons, we explored the use of BSM, given as a treatment for symptoms of antenatal depression, and its effect on infant temperament in the first year of life. This is the first study of its kind, and although there has been some investigation into the effect of *in-utero* micronutrient supplementation on infant development (83–86), influence on infant temperament has not been the main focus.

Across the three temperament dimensions assessed using the IBQ-R:VSF, the general trend over time within our sample was consistent between exposed and unexposed infants, with no significant differences, suggesting no adverse effects of *in-utero* BSM on infant temperamental outcomes in the first year of life. Given exposure to antenatal depression is associated with more negative displays of temperament, BSM-exposed infants may have been at greater risk of poorer outcomes; however, it appears *in-utero* exposure to BSM may mitigate the known risks associated with antenatal depression, as BSM-exposed infants displayed temperamental characteristics on par with typical pregnancies where symptoms of depression were not present.

The ORC unconditional model revealed a significant overall group decrease in orienting/regulatory capacity over time, and while this decrease may seem unexpected, given the general understanding that regulatory capacity increases over time as an infant develops, it is consistent with the effect of increased mobility. This increase in mobility as the infant ages likely leads to greater dissatisfaction with remaining stationary, and a growing desire for independence from a caregiver. This results in fewer behaviors associated with high loading on the ORC scale, typically seen more in younger infants, which contribute to the overall decreasing ORC score, e.g., measures of perceptual sensitivity, duration of orienting, cuddliness and low intensity pleasure.

The decrease in ORC observed in our results are consistent with existing literature suggesting that older infants are less likely to enjoy being held closely by a caregiver or be involved in quiet activities, have a preference for high intensity stimulation (16), possess increased ability to habituate to objects more rapidly and more control over attentional processes which allow infants to disengage from stimuli more efficiently as they develop (87).

Within our sample, higher gestational age at birth predicted smaller individual increases in positive affectivity over time. From a developmental perspective, it is purported that infants with an increased gestational age may have been marginally more developmentally advanced initially. Given this initial advantage, it appears these infants displayed a slower rate of growth compared to those of a lower gestational age, which results in the observed lack of longitudinal change.

The significant association between higher breastfeeding occurrences and negative affectivity is also consistent with previous literature (87, 88). Infants of breast-fed mothers have been identified as more irritable, displaying more negative affect and fussiness compared to mixed-fed and formula-fed infants (89–92). The increased displays of negative affect (e.g., crying and irritability) may stem from the perceived stress associated with mastering a successful latch for both mother and baby.

Given the role that temperament plays in the dyadic nature of the mother–infant relationship, every effort should be made to protect vulnerable infants from challenges that could arise related to social and emotional development. BSM appears to be a promising option given its success in treating antenatal depression (71), and the current study provides reassurance in its safety with relation to infant temperament, suggesting that by mitigating the risks associated with antenatal depression, we can set these potentially at-risk infants on a more positive developmental trajectory.

## 7 Strengths, limitations and future research

The present study involved a longitudinal multi-trait assessment of infant temperament across the first year of life. Using latent growth curve modeling, we could disentangle within- and between-child effects to closely examine whether BSM exposure predicted either higher initial levels or developmental changes in temperament. To our knowledge, this is the first study to examine how BSM supplementation during pregnancy relates to infant temperament. Antenatal depression is a known risk factor for a more difficult infant temperament, thus assessing the impact of a nutritional intervention used as a treatment for antenatal depression provides vital information on whether some of those risks can be mitigated.

Limitations included a modest sample size with likely underpowered statistical analyses which may explain the lack of significant findings. Future studies should sample a larger group of mothers to provide adequate statistical power to evaluate these research questions and include a more diverse population (e.g., ethnic minority, low socioeconomic status). Still, the findings from this study could be preliminarily informative, for instance, by examining the findings from a perspective focused on effect sizes rather than null hypothesis significance tests. Through this lens, the role of BSM exposure appears more positive: The three effects that met Cohen’s 1988 criteria for a “small” standardized beta (i.e., ≥ 0.10) (93) were all favorable: BSM exposure on longitudinal changes in orienting/regulatory capacity (*β* = 0.266) as well as on initial levels and longitudinal changes in negative affectivity (βs = −0.116 and − 0.235). The direction of these correlations indicate that BSM exposure does not negatively impact infant temperament in the first year of life and may exert a small positive influence.

Another potential limitation involved using mothers who had experienced antenatal depression as informants of their children’s temperament as maternal depression has been associated with informant discrepancies of children’s functioning (94). It is possible that mothers whose antenatal depression improved via BSM exposure tended to perceive and rate their children’s temperament more favorably than those whose depression did not improve at all or did improve but not to the same degree. Nevertheless, we expect that any effects of maternal depression would remain relatively stable across assessments and therefore be partial out by the intercept. This means that inter-individual differences in maternal depression would not account for the links between BSM exposure and within-infant changes in temperament over time [i.e., latent slopes; for a related discussion, see (95)].

Finally, there is existing evidence that maternal diet can impact temperament, thus it is plausible that diet may impact infant temperament differently to nutrient supplementation. Although information was collected on maternal nutritional risk based on dietary intake at study entry, the questionnaire only assessed nutritional risk based on food consumed within the past 7 days, thus is not appropriate to infer a dietary pattern across gestation.

## 8 Conclusion

Managing maternal mental health has become a central component in antenatal care, and a recent RCT showed that introducing a BSM regimen for pregnant women with antenatal depression could result in meaningful improvements in their mental health (71) in addition to positively influencing infant birth and neurobehavioral outcomes in the first weeks of life (71, 86). The current study investigated whether this BSM exposure had any impact on the temperament of these women’s infants across their first year of life. At the very least, our results indicate that BSM is effective in mitigating the risks associated with untreated antenatal depression, do not appear to increase any adverse risk to the infant temperament longitudinally, and may even indicate a small but positive effect.

## Data availability statement

The raw data supporting the conclusions of this article are not readily available as the participant’s consent has not been provided. The original MPlus data files are available. Requests to access these files should be directed to the corresponding author, julia.rucklidge@canterbury.ac.nz.

## Ethics statement

The studies involving humans were approved by Southern Human and Disabilities Ethics Committee (ref: 16/STH/187) and the Standing Committee on Therapeutic Trials (ref: 16/SCOTT/131). The studies were conducted in accordance with the local legislation and institutional requirements. Written informed consent for participation in this study was provided by the participants’ legal guardians/next of kin.

## Author contributions

SC: Conceptualization, Data curation, Formal analysis, Investigation, Methodology, Project administration, Writing – original draft, Writing – review & editing. SD: Formal analysis, Supervision, Writing – review & editing. JH: Conceptualization, Data curation, Methodology, Supervision, Writing – review & editing. HB: Conceptualization, Data curation, Methodology, Writing – review & editing. JR: Conceptualization, Data curation, Formal analysis, Funding acquisition, Investigation, Methodology, Supervision, Writing – review & editing.
